# *Angiostrongylus vasorum*: epidemiological, clinical and histopathological insights

**DOI:** 10.1186/s12917-014-0236-1

**Published:** 2014-09-28

**Authors:** Laura Rinaldi, Laura Cortese, Leonardo Meomartino, Teresa B Pagano, Paola Pepe, Giuseppe Cringoli, Serenella Papparella

**Affiliations:** Department of Veterinary Medicine and Animal Productions, University of Naples Federico II, Via Della Veterinaria 1, 80137 Naples, Italy; Inter-University Center for Research in Parasitology (CIRPAR), Naples, Italy

**Keywords:** *Angiostrongylus vasorum*, Dog, Pathology, Diagnosis

## Abstract

**Background:**

Canine angiostrongylosis is a nematode infection in domestic dogs and wild carnivores. The present report focuses on epidemiological, clinical and histopathological findings in a case of fatal disseminated angiostrongylosis in a dog living in southern Italy and provides data on the extent of the spread of *Angiostrongylus vasorum* in the same area.

**Case presentation:**

A 4-year-old female English Setter from the Campania region of southern Italy was referred with a 2-week history of cough and severe respiratory distress that did not respond to antimicrobial therapy. Based on clinical, radiological, echographical and cytological findings (including the presence of larvae), a suspect diagnosis of lungworm infection was performed. After few days the dog died due to progressive clinical aggravation. Complete postmortem examination was conducted within 24 hours from death and samples from lungs, heart, liver, kidney, spleen, stomach and small intestine were fixed in 10% buffered formalin. Grossly, several hemorrhagic foci were observed mostly in the lungs, liver, kidney. Microscopically, the lungs contained numerous, multifocal to coalescing granulomas composed of epitheliod macrophages, multinucleated giant cells and some neutrophils, frequently associated with parasite eggs and larvae. The lungs contained many firm nodules, many adult nematodes approximately 1.5 to 2 cm in length were observed in cut sections and identified as *A. vasorum*. A subsequent parasitological survey performed with FLOTAC on stray dogs living in the same area showed the presence of *A. vasorum* larvae in 17 of 1639 stray dogs examined (1.04%).

**Conclusion:**

This survey provides new data on distribution of *A. vasorum* and underlines that canine angiostrongylosis should be considered as differential diagnosis in dogs.

## Background

*Angiostrongylus vasorum* commonly known as the “French heartworm” is a metastrongyloid nematode of dogs and other canids, including various species of fox, wolf, coyote and jackal (definitive hosts) [1]. The adults reside in the right side of the heart and in the pulmonary arteries, with potentially severe consequences for the host [2]. The infection can cause a wide range of disease outcomes, which are most often characterized by respiratory dysfunction, but it can also manifest as bleeding, neurological, cardiovascular or gastrointestinal disorders, with or without respiratory involvement [3].

The geographic distribution of the parasite includes various countries of Europe, North and South America as well as Africa [1]. The parasite appears to be quite common in well-isolated endemic foci, with few occasional reports occurring outside them [4]. However, recent reports challenge this traditional view, as *A. vasorum* has appeared in several new geographical areas [5]. Italy is one of the countries where *A. vasorum* is undoubtedly spreading, offering ideal environmental and epidemiological conditions for the expansion of *A. vasorum* and the establishment of further new endemic foci [6,7]. The reasons for this emergence are unclear and poorly understood, but may involve global changes leading to spread of a variety of definitive, intermediate and paratenic hosts and consequent modification of mollusk phenology [8]. Due to this spread and the increasing clinical relevance of canine angiostrongylosis, the interest in *A. vasorum* is growing, particularly with respect to its treatment and control [9].

At present, diagnosis relies on clinical manifestations, diagnostic imaging, bronchial washings and on the detection of the first-stage larvae (L1) in faecal samples. The parasite detection in faecal samples is usually performed using conventional coprological examination such as direct faecal smears, flotation and Baermann (gold standard) [10]. Also, the FLOTAC techniques [11] have been demonstrated as very sensitive for the diagnosis of *A. vasorum* in dogs [12]. Furthermore, serological [13,14] and molecular [15,16] methods have been developed for the diagnosis of canine angiostrongylosis.

With the aim to provide further insights on this parasite, the present paper describes *post mortem* gross and histopathological observation in a dog with a fatal infection by *A. vasorum*. Subsequently to this case, a coprological survey was performed to evaluate the extent of *A. vasorum* infection in stray dogs living in the city of Naples (southern Italy), using the FLOTAC techniques.

## Case presentation

A 4-year-old female English Setter from the Campania region of southern Italy was referred with a 2-weeks history of cough and severe respiratory distress that did not respond to antimicrobial therapy. On physical examination, the dog was alert, dyspneic and tachypneic. Mucous membranes and capillary refill time were normal. Cardiac auscultation revealed tachycardia.

Hematological abnormalities included anemia and leukocytosis. Routine biochemical analyses were unremarkable. An electrocardiogram demonstrated right axis deviation. Thoracic radiographs in standard lateral-lateral projection and toracic ultrasound were carried out and an ultrasound-guided fine needle aspiration was performed on selected pleural lesions. Cytological evaluation showed mixed inflammatory cells occasionally admixed with full nematode larvae. Based on clinical, radiological, ultra sonographical and cytological findings (including the presence of larvae), a suspect diagnosis of lungworm infection was made; however, no larvae were detected in faecal samples using the Baermann technique [17]. A therapeutic protocol including antibiotics, corticosteroid and milbemycin oxime was administered. Ten days later the dog was hospitalized following a severe pneumothorax. Supplemental oxygen therapy was administered and a chest tube was placed. Subsequently pneumothorax developed again; thoracic surgery was necessary and led to its resolution. One month after surgery, the dyspnea worsened. After a few days, the dog suddenly died despite supplemental oxygen therapy. Complete postmortem examination was carried out within 24 hours of the death and samples from lungs, heart, liver, kidney, spleen, stomach and small intestine were fixed in 10% buffered formalin. Pulmonary arteries were opened and examined in order to identify intravascular nematodes. Some cytological smears were performed from tracheobronchial lymphnode. Many adult worms were collected and morphologically identified as adult *A. vasorum* based on morphology of the adult male bursa and the length of the spicules [18]. Formalin-fixed samples were processed for sectioning, embedded in paraffin, sectioned at 5 micron, and stained with hematoxylin and eosin (HE), and Periodic Acid Shiff (PAS) stain.

### Radiological findings

**Figure 1:**
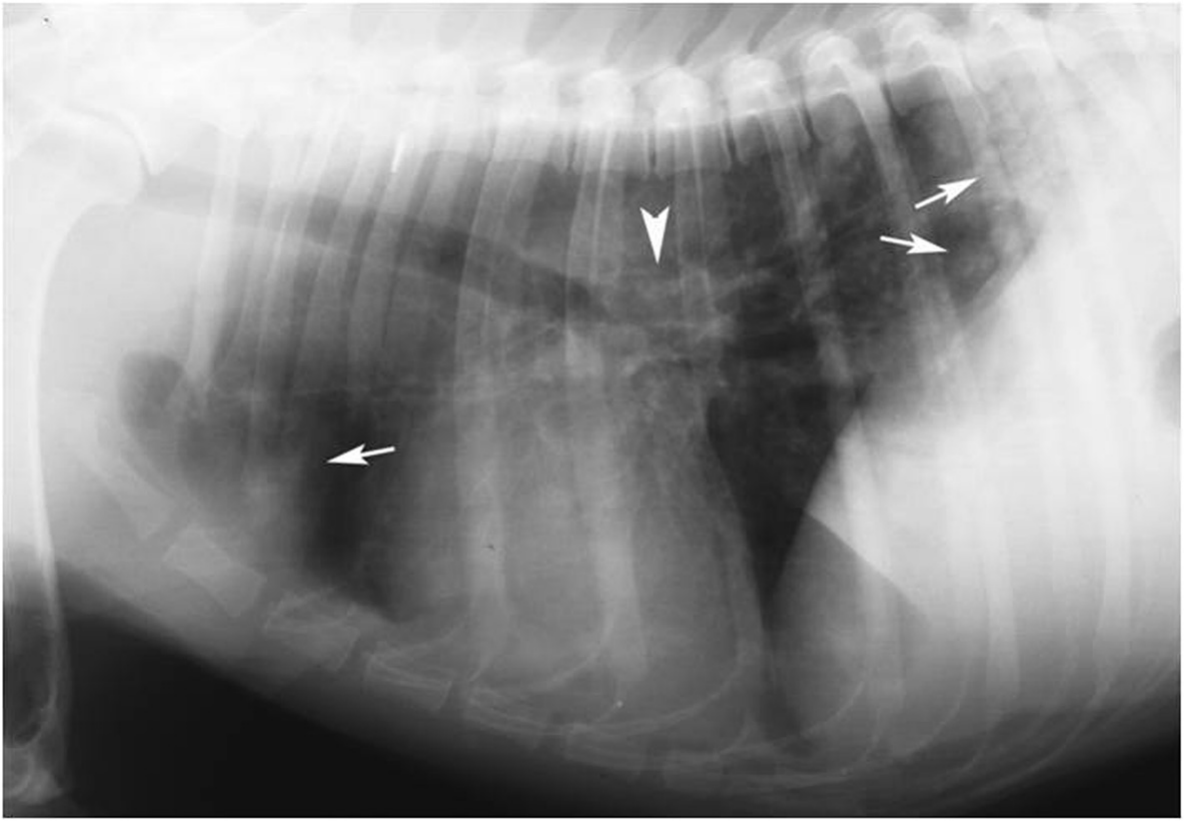
**Lateral radiograph on right recumbency: there is a diffuse opacification on lung fields with a mixed nodular interstitial, bronchial and alveolar pattern (arrows); tracheobronchial lymph nodes are enlarged (arrowhead).**

**Figure 2:**
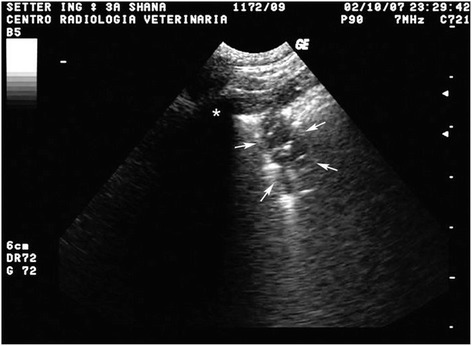
**Ultrasound dorsal longitudinal scan trough the 9th left intercostal space: a sub pleural hypoecoic nodule is visible (arrows); residual air in alveoli and bronchioles appears as hyperecoic speckles inside the nodule; on the left side there is a rib acoustic shadowing (asterisk).**

Thoracic radiographs showed a multifocal to coalescing bronchial and alveolar pattern and enlargement of tracheobronchial lymph nodes (Figure 1). Thoracic ultrasound showed multiple sub pleural nodules (Figure 2) from which an ultrasound-guided fine needle aspiration was performed. B-mode echocardiography showed a mild dilation of the right atrial, main pulmonary trunk and right pulmonary artery. Doppler echocardiography revealed tricuspid regurgitation (maximal velocity: 243 cm/s; maximal pressure gradient, 23,6 mmHg).

### Pathological findings

**Figure 3:**
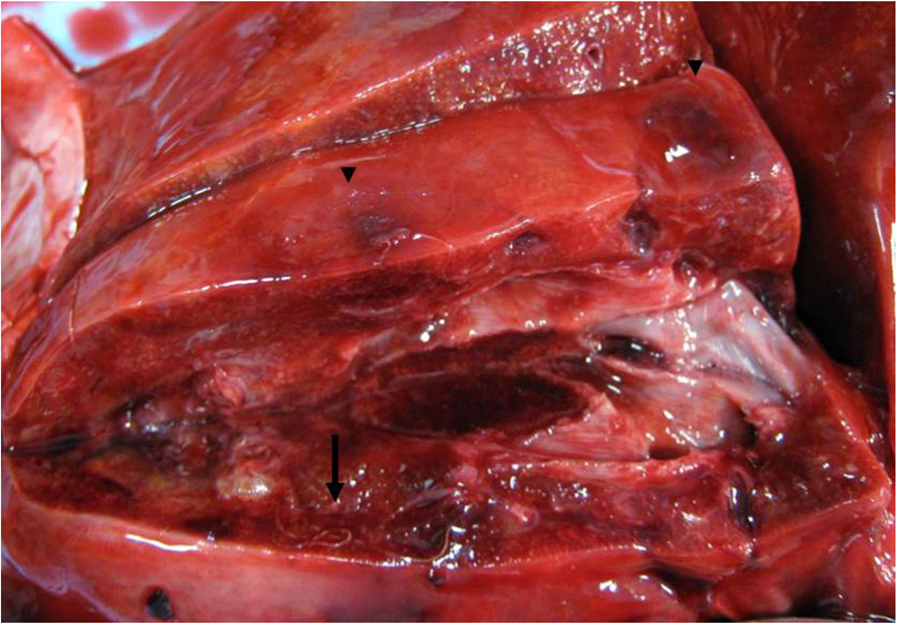
**Lung.** Pulmonary inflammation and fibrosis. Note the presence of necrotic hemorrhagic areas (arrowheads) and some larvae (arrow).

Macroscopically, pathological findings of the lungs consisted in multiple, poorly defined dark-red foci of necrotic-hemorrhagic areas. Firmer areas of broncopneumonia were also present. Several adult parasites, approximately 2–2.5 cm in length, were collected from pulmonary arteries (Figure 3). Alveolar septa were mildly expanded with an increased amount of dense fibrous tissue. The overlying pleura were moderately thickened too.

**Figure 4:**
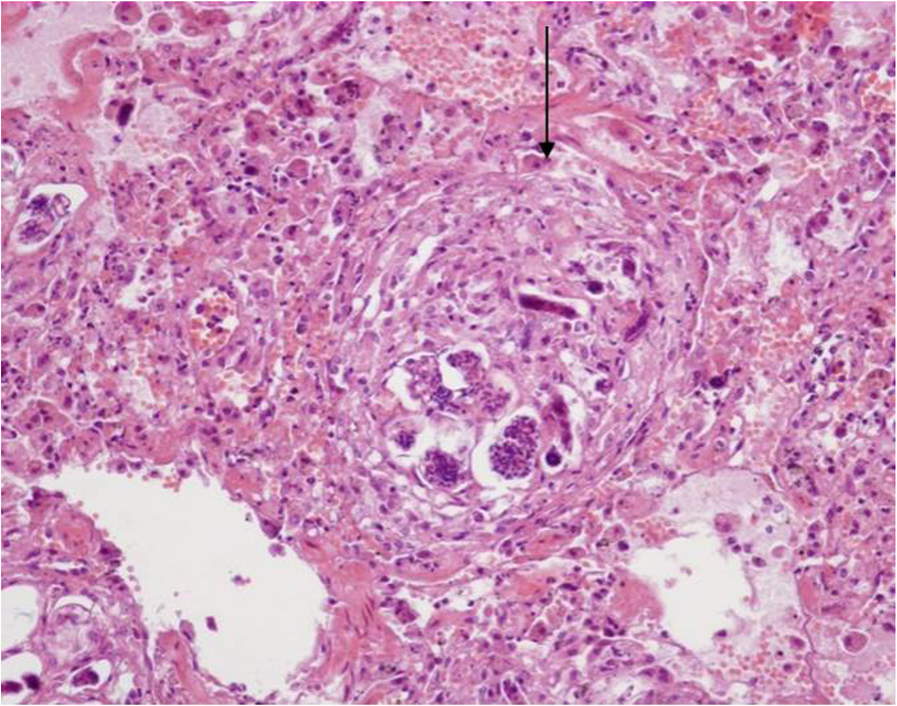
**Lung.** Pulmonary granuloma (arrow). Some unembryonated and embryonated parasite eggs, coiled larvae and cellular debris are visible in the center. H & E stain (20×).

**Figure 5:**
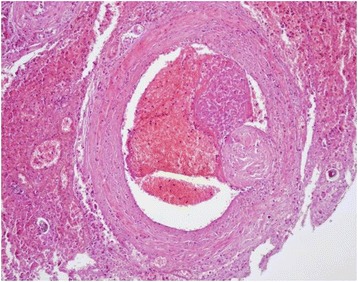
**Lung.** Large organized thrombus in lumen of an artery. H & E stain (10×).

Microscopically, large areas of pulmonary parenchyma were replaced by numerous, multifocal to coalescing granulomas composed of macrophages, multinucleated giant cells, eosinophils and neutrophils granulocytes. The center of these granulomas contained unembryonated and embryonated parasite eggs, coiled larvae and deposits of amorphous, pale, eosinophilic necrotic debris, while lymphocytes and plasma cells were present at the periphery (Figure 4). Fibroblastic proliferation with septal thickening and lymphoid hyperplasia were also observed. Numerous organized thrombi (Figure 5), often containing large adult nematodes were present in lumen of medium-sized pulmonary arteries, causing eosinophilic vasculitis. Occasionally, adult parasites were seen within the alveoli, which were affected by productive alveolitis with cell esfoliation and presence of luminal suppurative exudate. Wide hemorrhagic areas associated with hemosiderin deposits, hemosiderin-laden macrophages, coagulative necrosis foci, edema and hypersecrection of mucous glands where found in the remaining parenchyma.

Many adult worms were found in right ventricular cavity. Macroscopically, the right ventricular wall was thickened and trabeculae carnae muscles were hypertrophied. The whole heart appeared moderately enlarged and rounded.

**Figure 6:**
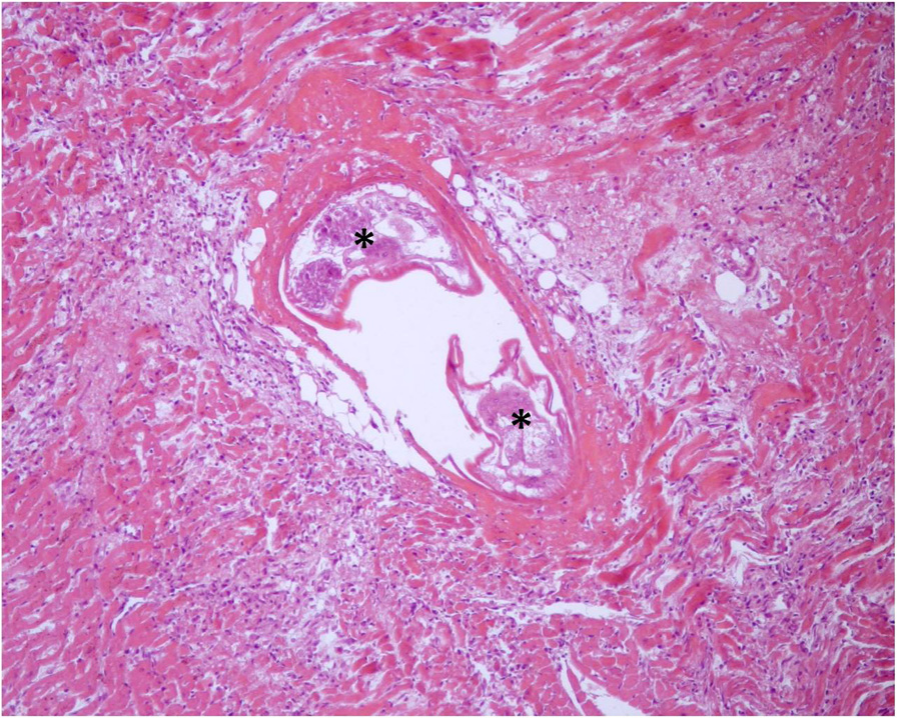
**Heart.** Adult worms in vessel lumen (asterisks), inducing vasculitis and perivascular inflammation. Note also the coagulative necrosis of the cardiomyocytes and interstitial myocarditis. H & E stain (10×).

**Figure 7:**
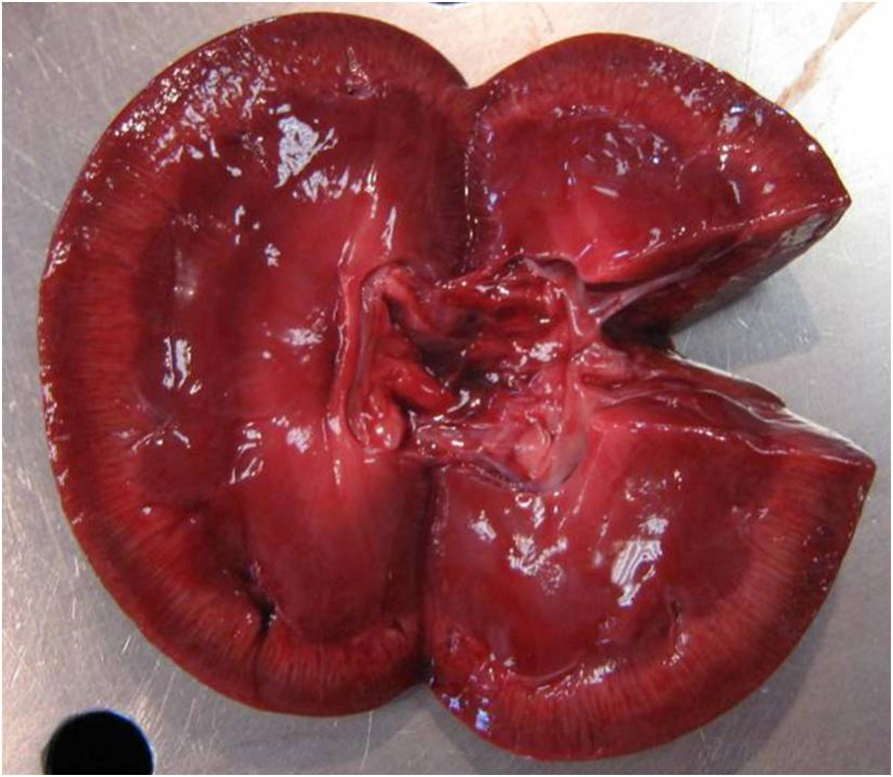
**Kidney.** Interstitial nephritis.

Microscopic myocardial injuries resulted from aberrant larval migration; multifocally, granulomatous foci mainly centered around parasite eggs or larvae were detected, disrupting the normal myocardial architecture. Multiple foci of necrosis of cardiomyocytes associated to interstitial edema and infiltration of eosinophils was observed as well. Occasionally, adult worms were found in vessel lumen, inducing vasculitis and perivascular inflammation (Figure 6). Grossly moderate bilateral enlargement of the kidneys was observed; radial, whitish striae were evident on the cut surface (Figure 7).

**Figure 8:**
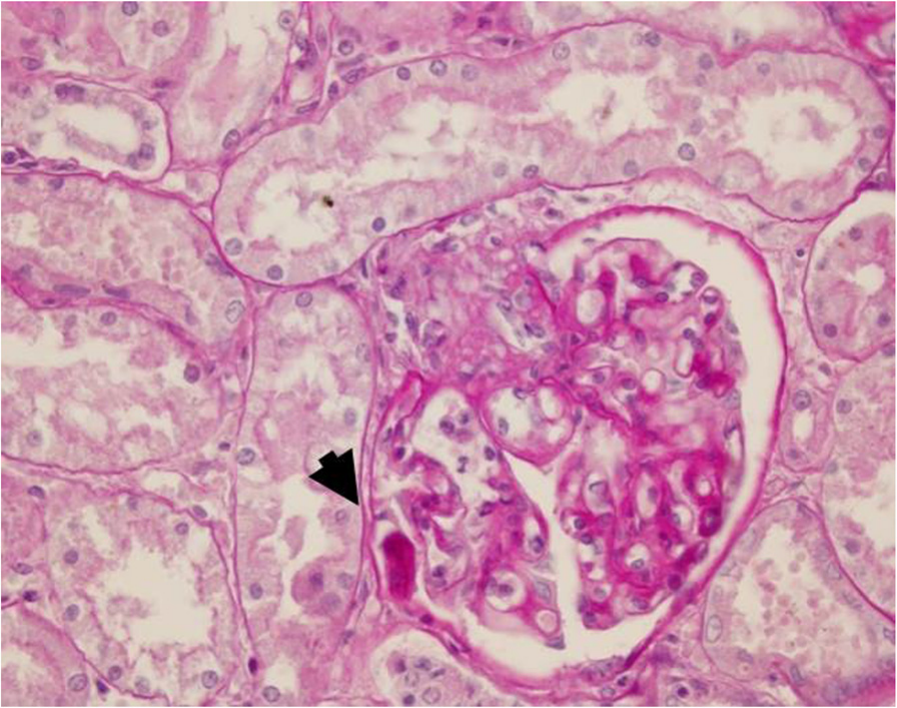
**Kidney.** Membranoproliferative glomerulonephritis: apparent duplication of the glomerular basement membrane (tram-track appearance) S. A larva within the glomerulus is also visible (arrowhead). PAS stain (40×).

Histologically, several renal injuries were present. PAS stain demonstrated membranoproliferative glomerulonephritis with apparent duplication of glomerular basament membrane (tram-track appearance) was observed (Figure 8), thickening of Bowman’s capsule and glomerular mesangial matrix deposits. Glomerular atrophy, thrombi and larvae within the glomerular capillaries were also reported. Other histopathological findings included hyaline droplet nephropathy, interstitial inflammatory infiltrates, and hemorrhages.

In the liver numerous single, disseminated inflammatory elements, consisting of lymphocytes, plasma cells and eosinophil granulocytes were observed either in the portobiliar spaces or around centrolobular veins. Some small granulomas containing the remains of larvae were also present. The remaining hepatic parenchyma showed moderate interstitial hemorrhages, deposits of bile pigments and focal hyperplasia of Kupffer’s cells.

The superficial mucosa and the deep *lamina propria* of the stomach showed several clusters of mononuclear inflammatory cells admixed with eosinophil granulocytes. Sometimes the inflammatory infiltrate was perivascular.

Signs of eosinophilic enteritis, mostly involving the small intestine, were observed. They included lymphoid hyperplasia, presence of numerous eosinophil granulocytes and mononucleated inflammatory cells in the axis of the villi and hypersecrection with cystic aspects of the mucous glands. This inflammation remained confined at the *lamina propria* level, without involving the lower layers of the intestinal wall.

Microscopically, the pancreas showed interstitial hemorrhages, focal eosinophilic inflammatory infiltrates and some small granulomas, which contained nematode eggs or larval remains.

The spleen were affected by a severe lymphocytic depletion, while the connectival septa and the serosa appeared very thickened.

### Coprological survey

Between January 2012 and December 2013 faecal samples were routinely collected from 1639 stray dogs living in the city of Naples (Campania region, southern Italy) and brought to the veterinary hospital of the Department of Veterinary Medicine for sterilization. Information regarding sex, age and clinical signs was collected at the time of arrival to the veterinary hospital. The age of each animal was recorded based on dental examination. A minimum of 2 g of faeces was collected from each animal, immediately placed into a container, and fixed 1:4 with formalin 5% before being analyzed.

**Figure 9:**
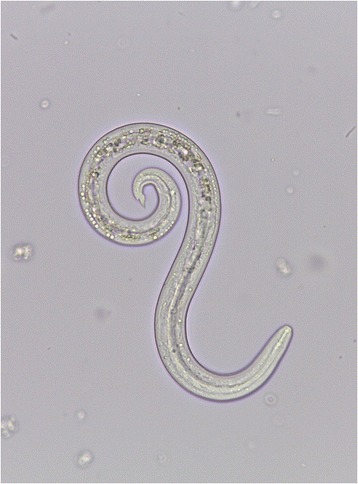
**First-stage larva of** ***Angiostrongylus vasorum*** **.** The L1 larvae when examined under a light microscope are approximately 350 μm in length (range 310–400 μm), with a characteristic kinked tail and a dorsal spine and notch.

Each sample was examined by the FLOTAC basic technique [12] (using zinc sulphate s.g. 1.20 as flotation solution). A differential diagnosis was also performed to discriminate *A. vasorum* first stage larvae (L1) from those of *Crenosoma vulpis*, *Oslerus osleri* or *Filaroides* spp. on the basis of morphological finding at the tail according to Helm et al. [19]: the tapered tip of the tail of *A. vasorum* L1 has a kink with a dorsal spine (Figure 9).

The presence of *A. vasorum* was detected in 17 of 1639 stray dogs examined (1.04%; 95% CI = 0.63-1.69). Among the others lungworms, only *O. osleri* was detected in 2 of the 1639 dogs examined (0.12%; 95% CI = 0.02-0.49).

## Conclusions

This report documents a case of fatal canine angiostrongylosis in southern Italy. The clinical, pathological, and parasitological findings are consistent with a severe and diffuse angiostrongylosis. In fact, the clinical manifestations associated with this disease may vary greatly from subclinical state (with no or minor sign) to fatal condition. The cardiorespiratory distress due to the inflammatory response to eggs and migrating larvae [20,21], bleeding disorder such as hemorrhagic diatheses and coagulation defects, cardiac failure and other nonspecific miscellaneous systemic and gastro-enteric signs such as gagging, coughing, vomiting, oedema, anorexia, weight loss, stunted growth as well as decreased exercise tolerance are the most common clinical symptoms [1,22,23]. As described above, the definitive diagnosis requires demonstration of the first stage larvae in the faeces, tracheal wash or bronchoalveolar lavage. Several case reports, including this one, show that faecal Baermann examination can be false negative, due to intermittent shedding of larvae, a high variation in the number of shedded larvae and the long pre-patent period [2]. Thus, examination of faecal samples from three consecutive days is recommended and tracheal wash should be considered [2].

The respiratory syndrome observed in the *antemortem* case is typical of that reported for *A. vasorum* infection in dog. The pulmonary lesions in dog angiostrongylosis are well described in the literature [24] and the present findings are in accordance with current knowledge [25]. The most consistent finding in our dog was interstitial pneumonia with prominent vascular changes. Pneumonia, in this case, was generally granulomatous with variable amounts of suppurative and eosinophilic inflammation. Small granulomatous foci of inflammation, associated with nematodes eggs and larvae, were found in many organs such as kidney, liver, pancreas. This is in accordance with the recent findings that showed the presence of the lesions in different organs [2], not only in the cardiovascular and respiratory systems. Thus, the absence of the typical signs should not preclude consideration of angiostrongylosis as a differential diagnosis [26]. The pathological findings in our case are in according with this propensity and should be considered during the diagnosis of suspected cases.

The findings of the present study confirm that the French heartworm *A. vasorum* should be considered in the differential diagnosis of pulmonary disease in dogs in Italy [27].

The results of this research demonstrated also the occurrence of *A. vasorum* L1 in fecal samples of dogs from the Campania region, as reported in other areas of central and southern Italy with an infection rate similar to that recently reported also in other studies [28-32]. In addition, they also show that FLOTAC can be utilized for diagnosis of *A. vasorum* infection, as already demonstrated for other lungworms as *Crenosoma vulpis* [33] in dogs and *Aelurostrongylus abstrusus* [34] in cats. Furthermore, given the lack of specificity of clinical signs, these infections are often not included in differential diagnosis, and animals remain infected and untreated. The FLOTAC has the advantage to be multivalent and therefore also other parasites (protozoa, nematoda, trematoda and cestoda) can be detected, which may be important in determining the cause of non-specific symptoms. A valid and affordable diagnostic method for the detection of *A. vasorum* – infected animals before the appearance of clinical signs could avoid the onset of severe pathological changes in early anthelmintic-treated animals [35].

The emergence of *A. vasorum* as an important agent of respiratory disease in dogs, and apparent ongoing expansion of its range, must underline the need of an enhanced awareness concerning the knowledge of its epidemiology and biology [4].

### Ethics

The animals used in the present study were sampled following approval by the animal ethics and welfare committee of the University of Naples Federico II (in Italian, Comitato Etico-scientifico per la Sperimentazione Animale dell’ Università di Napoli Federico II; protocol number 0075262).
